# Molecular Structure, Theoretical NBO Analysis, Vibrational Spectrum of CO_2_-Responsive Hydroxyamidine-Based Ionic Liquid: A Combined Computational and Experimental Approach

**DOI:** 10.3390/molecules31061055

**Published:** 2026-03-23

**Authors:** Lyazzat Abulyaissova, Nikolay Barashkov, Irina Irgibaeva, Yerbolat Tashenov

**Affiliations:** 1 Chemistry Department, Buketov National Research University, Karaganda 100028, Kazakhstan; 2 Micro-Tracers, Inc., 1370 Van Dyke Avenue, San Francisco, CA 94124, USA; 3 Department of Chemistry, L.N. Gumilyov Eurasian National University, Astana 010008, Kazakhstan

**Keywords:** carbon dioxide capture, hydroxyamidines, polymeric ionic liquid, intramolecular hydrogen bond, intermolecular contacts, absorption spectra, density functional theory

## Abstract

The utilization and chemical transformation of carbon dioxide remains a pressing problem in modern chemistry. Numerous experimental and theoretical studies have focused on the interaction of CO_2_ with amines. In this work, quantum chemical density functional theory (DFT) calculations of equilibrium geometries, energies, electronic and vibrational characteristics of CO_2_-sensitive mono-, di-, tris-hydroxyamidines and their associates were carried out by the B3LYP/6-31G(*d*, *p*) method. The harmonic vibrational frequencies were scaled and compared with the experimental FTIR spectra for supporting wavenumber assignments. Natural bond orbital (NBO) analysis of the atomic charges and charge delocalization was employed to investigate the nature of hydrogen bonding in hydroxyamidine associates. We also used the intrinsically polarizable continuum model (IEFPCM), and the DFT-D3 method was applied to account for dispersion effects during associate formation. Using the 6-311+G(2*d*, *p*) basis set for tris-hydroxyamidine, and its adducts, a comparative analysis of the experimental and calculated ^1^H NMR spectra was performed. Here, we considered non-trivial sites of carbon dioxide absorption and hydroxyamidine protonation, which, to our knowledge, have hardly been considered by other authors. Present DFT results agree rather well with the experimental data and support new insight into the formation of the PIL structure.

## 1. Introduction

Carbon capture, utilization, and storage (CCUS), including carbon dioxide capture and storage (CCS), has been and remains a key scientific and technical challenge. High atmospheric CO_2_ concentrations pose global environmental challenges.

However, through continuous photosynthesis and carbon dioxide absorption in plants, carbon dioxide levels in the atmosphere are self-regulated. So, increasing plant cover is one of the most effective ways to maintain ecological balance. Another promising approach is the incorporation of carbon dioxide into closed technological cycles, which should also contribute to reducing the greenhouse effect.

Advanced carbon dioxide capture technologies have been described in the literature, but they have both advantages and disadvantages from a technical standpoint [1,2,3,4,5,6,7,8]. One traditional and accessible method of CO_2_ capture is chemical absorption with aqueous amine solutions, which has been the subject of numerous applied and theoretical studies [9,10,11,12,13,14,15,16,17,18,19,20,21,22]. However, the use of amines is not without its drawbacks: high energy consumption, the formation of hazardous nitrosamines, amine losses, etc. Therefore, the search for new types of more effective absorbents/adsorbents continues.

Some promising candidates are amidines [23,24,25,26,27]. In [23], a study of the interaction of the polyamidine-polyethylene glycol binary system with carbon dioxide showed that 66% of the amidine groups bind CO_2_ molecules. The authors of [24] obtained new polyamidine polymers capable of selective and reversible carbon dioxide capture at room temperature and under atmospheric pressure. The monomer 4-vinylbenzyl amidine, which changes its hydrophobicity/hydrophilicity under the influence of CO_2_, was developed and synthesized [25]. We previously reported on the possibility of using carbon dioxide gas to obtain polymeric ionic liquids (PILs) by interaction with mono- and tris-amidines [26,27].

The mechanism of interaction of carbon dioxide with the listed and other compounds is studied both experimentally and using computer modeling methods. In order to fundamentally understand the mechanism of CO_2_ capture by unhindered and hindered amines, the authors of [9] performed density functional theory calculations that revealed opposite kinetic results for different amines, namely, that the formation of bicarbonate for 2-amino-2-methyl-1-propanol (AMP) is more favorable than the formation of carbamate. However, the situation is reversed for monoethanolamine (MEA). This explains well the experimental observation of different product distributions in the reactions of AMP and MEA with CO_2_. According to the authors, the difference in the electrostatic potential distribution of AMP and MEA is a possible reason leading to the different mechanisms of their reactions.

The study [10] discusses the formation of the nitrogen (monoethanolamine)–carbon (CO_2_) bond under solvent conditions based on molecular theory using RISM-SCF-SEDD, a hybrid method of quantum chemistry of the solute and statistical mechanics of the solvent. Authors clarified that the role of amine as a base is crucial in the proton transfer step. In [14], the thermochemistry of carbamate and bicarbonate formation was calculated using the composite CCSD(T) and DFT methods. Carbon dioxide chemisorption was shown to occur via the carbamate intermediate for all primary and secondary amines. To comprehensively understand the mechanism of carbon dioxide absorption by aqueous amine solutions, the authors of [15] performed ab initio molecular orbital calculations in combination with the solvation model (PCM). Based on the calculated activation energies, an efficient pathway for the direct interconversion of carbamate and bicarbonate without the participation of free CO_2_ was proposed.

DFT-B3LYP modeling [18] was used to characterize the products and energetics of reactions of carbon dioxide with a number of substituted amines at amine/CO_2_ stoichiometry of 1:1 and 2:1. The results showed that by controlling both the nature and arrangement of functional groups, the reaction energies can be tuned over a significant range. A quantum chemical analysis of carbon dioxide absorption by aqueous amine solutions using the continuum solvation model (SMD/IEF-PCM) in combination with density functional theory (DFT) was performed in [19]. Based on the obtained results, it was suggested that the carbamate anion forms by a two-step reaction via a zwitterion intermediate, and this occurs faster than the formation of the bicarbonate anion.

Unique and unexpected reaction mechanisms involved in CO_2_ absorption by aqueous hydrazine were revealed using ^1^H, ^13^C and ^15^N NMR spectroscopy in combination with first-principles quantum mechanical modeling [20]. In [21], the reaction pathways of carbamate as well as bicarbonate formations were analyzed by DFT modeling. The authors of [22] considered the formation of carbamate, carbamic acid and bicarbonate in the reaction of carbon dioxide with a sterically hindered amine and an aromatic amine based on molecular dynamics calculations.

A literature survey reveals that many of them are devoted to the interaction of carbon dioxide with amines, particularly under aqueous conditions. As an alternative to amines, amidines have also attracted the attention of researchers, as discussed above. Take our previous work as an example; we also studied the interaction of CO_2_ with mono- and tris-amidines to obtain polymeric ionic liquids (PILs) and proposed the structure of the PILs [26,27].

So, conventional CO_2_ capture systems are typically based on amine–CO_2_ chemistry, where absorption proceeds via carbamate or bicarbonate formation in aqueous media and is governed by carbonic acid equilibria [28,29,30]. Alcohol-based systems may additionally form alkylcarbonate species upon reaction with CO_2_ [31]. In contrast, the hydroxyamidine systems studied here exhibit a distinct binding mechanism: CO_2_ uptake occurs through hydroxyl-containing amidines, forming alkylcarbonate-type zwitterionic structures stabilized by hydrogen bonding. Moreover, methanol present in the reaction medium may participate in a proton-relay mechanism facilitating proton transfer between the carbonate fragment and the imino nitrogen atom. This pathway differs from classical carbonic-acid-mediated CO_2_ capture and provides new insight into CO_2_ fixation in alkylhydroxy-functionalized amidine systems.

In this work, which continues our research, we synthesized dihydroxylamidine with subsequent CO_2_ treatment, then modeled and investigated the structure of three hydroxyamidine-based PILs containing mono-, di-, tris-hydroxyl groups and the interaction of CO_2_ with hydroxyamidines using a combined experimental and computational approach. Here we (a) use quantum chemical calculations using the density functional theory (DFT) method, and conduct a comparative analysis of previously obtained and present experimental infrared absorption and ^1^H NMR spectra with the calculated spectra; (b) consider associates in the absence of water molecules, but in the presence of methanol as a medium.

## 2. Results and Discussion

### 2.1. Molecular Geometry of Hydroxyamidines

**Figure 1:**
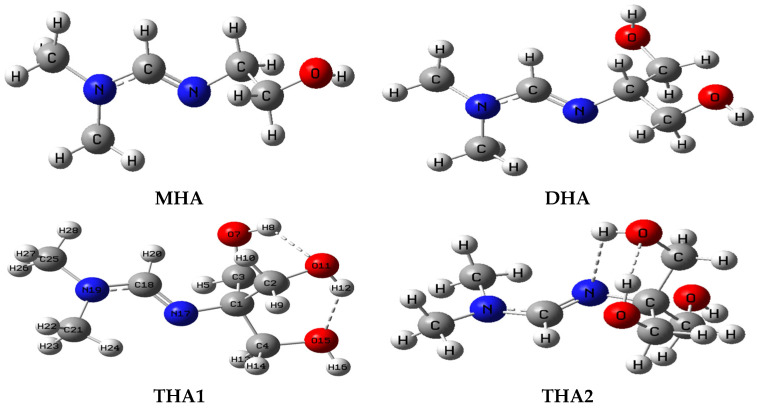
Geometric structures of mono-, di- and tris-hydroxyamidine molecules in the ground electronic state according to the B3LYP/6-31G(*d*, *p*) method (atom numbering according to the GaussView program).

Stable conformations of hydroxyamidine molecules (Figure 1) contain the functional group >N-C(R=H)=N- (amino and imino nitrogen) and hydroxyl groups: from one (monohydroxyamidine, MHA) to three (tris-hydroxyamidine, THA). Dihydroxyamidine (DHA) contains two hydroxyl groups. Optimized molecular structures of hydroxyamidines are shown in Figure 1; their energies taking into account zero-point vibrations are presented in Table 1. The structural parameters calculated for the equilibrium geometry of hydroxyamidines are given in Supplementary Table S1.

Figure 1 shows two conformations of tris-hydroxyamidine, THA1 and THA2, which differ in mutual arrangement of hydroxyl groups, the number of hydrogen bonds and the possibility of forming an intramolecular H-bond with the nitrogen atom. According to calculations, THA1 is 3.25 kcal/mol more energetically stable than THA2. In the tris-hydroxyamidine THA1 molecule, three OH groups form two intramolecular hydrogen bonds with internuclear O…H distances of 2.168 and 1.989 Å, which contribute to the energetic stabilization of the molecule. The latter are analogous to the distances in diols [32].

In the THA2 conformation, the only hydrogen O-H…O bond with an oxygen-hydrogen distance of 1.946 Å occurs between the two OH groups, but there is also the possibility of intramolecular H-bonding with the imino nitrogen. The consideration of a less stable conformation, THA2, can be justified by the fact that thermal fluctuations result in a statistical distribution of molecular conformations. Therefore, at a given temperature, molecules in various conformations are always present in the bulk of a substance, not just those in the conformation with the lowest energy. It should also be noted that molecules of the same compound in different phase states can differ in conformation; i.e., an energetically favorable conformation in one phase may be unstable in another. Biphenyl is noteworthy in this regard. Its molecule is planar in the crystal, but non-planar in the gas phase and isotropic solution—the phenyl rings are arranged at angles of 40–45° and 30°, respectively [33].

### 2.2. Correlation of IR Spectra with the Structure of Amidines

The molecular structures presented above contain several functional groups, which leads to the occurrence of absorption of radiation of the characteristic frequency. The influence of the rest of the molecule usually does not exceed 5%. The calculation of vibrational IR spectra was carried out only for structures minimized by potential energy in order to avoid forces of atom–atom interactions that are too large, leading to the appearance of negative frequencies in the spectrum. Quantum chemical calculations made it possible to obtain spectral characteristics of molecules, such as frequencies and forms of normal vibrations, as well as the intensities of IR bands for the entire spectrum region, including the low-frequency region (˂400 cm^–1^). However, due to the introduction of the adiabatic approximation in quantum chemical methods, a discrepancy is always observed in the calculated and experimental frequencies: the first, harmonic frequencies are usually overestimated compared to the experiment. In this regard, a scaling factor (0.95) was applied to adjust the calculated data to the frequencies calculated at the B3LYP/6-31G(*d*, *p*) level [34]. The scaled wavenumbers of the IR spectra of the structures are presented in Table 2.

**Figure 2:**
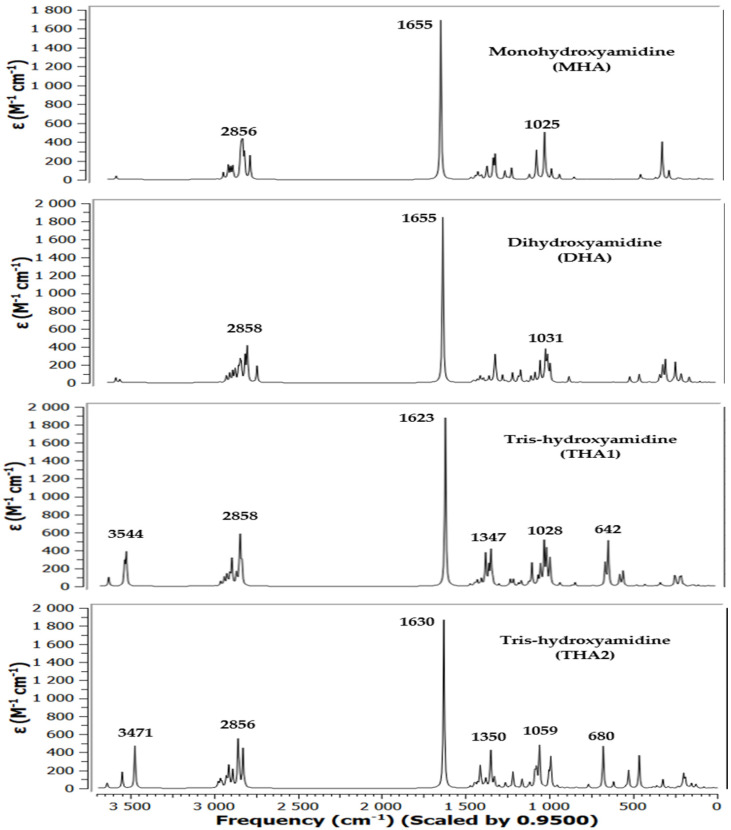
Calculated IR spectra of hydroxyamidine molecules (B3LYP/6-31G(*d*, *p*) method).

**Figure 3:**
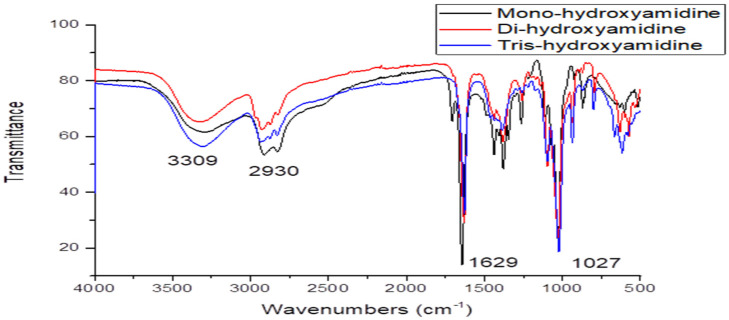
Experimental IR spectra of hydroxyamidines ([27] and this work).

To assign all bands in the IR spectrum of hydroxyamidines, a comparative analysis of the results of quantum chemical calculation of the vibrational absorption spectra of the molecules and the experimental spectra of the MHA, DHA and THA was performed. Figure 2 and Figure 3 show the theoretical spectra (in coordinates *absorption (transmittance) coefficient*, M^−1^ × cm^−1^—*wave number*, or *frequency*, cm^−1^) of four molecules and experimental spectra (in coordinates *transmission intensity*, %—*wave number*, cm^−1^) [27] and the present work’s IR spectra of three molecules, respectively.

#### 2.2.1. O-H Bonds

In the experimental IR spectra of mono-, tris- [27] and dihydroxyamidine (this work) compounds, vibration signals are observed in the region of 3300–400 cm^−1^.

The absorption bands of hydroxyl groups bound by hydrogen bonds usually lie in the region below 3550 cm^−1^, while free, unassociated ones are in the region of 3580–3650 cm^−1^. In the theoretically calculated spectra of three hydroxyamidine molecules, weak bands with frequencies in the region of 3619–3650 cm^−1^ and 3540–3555 cm^−1^ can be assigned to the stretching vibrations of unassociated OH groups and of intramolecular hydrogen bonded hydroxyl groups in the THA1 molecule, respectively (Table 2).

The absence of such signals in the experimental spectra indicates that there are no free OH groups in the molecules, and all the hydroxyl groups of hydroxyamidines participate in the formation of hydrogen bonds: intra- and/or intermolecular, it is also possible that some of the vibrations in the experimental spectra have low IR intensity. However, in the observed spectra of mono-, di- and tris-hydroxyamidines, bands of medium intensity are observed at 3294, 3319 and 3309 cm^−1^, respectively, which were attributed to the OH stretching vibration ([27] and this work). As has already been stated above, it is obvious that these are the associated OH groups.

#### 2.2.2. C-H Bonds

The absorption bands *ν_exp_* 2912 and 2827 cm^−1^, 2930 cm^−1^, 2936 and 2828 cm^−1^ of MHA, DHA and THA, respectively, belong to the stretching vibrations of C-H bonds of methylene and/or methyl, methine groups ([27] and this work).

In the theoretical spectra of the molecules, the absorption region in the range of 3010–2800 cm^−1^ corresponds to asymmetric and symmetric vibrations of C–H of the indicated groups (Table 2).

#### 2.2.3. C=N Bonds

One of the most intense bands in the experimental IR spectra of mono- (*ν_exp_* 1644.89 cm^−1^), di- (*ν_϶ĸcn_* 1644.89 cm^−1^), and tris-hydroxyamidine (*ν_exp_* 1629.93 cm^−1^) is due to the stretching vibrations of the C=N bond, which are observed in the theoretical spectra at 1654.70 and 1622.78 cm^−1^, respectively. In the spectrum of dihydroxyamidine, the corresponding vibrations appear at *ν_theor_* 1655.21 cm^−1^.

#### 2.2.4. C-O Bonds

Asymmetric and symmetric stretching vibrations of C-O bonds appear with very high intensity in the region of *ν_theor_* 1025 cm^−1^ for mono- (*ν_exp_* 1030 cm^−1^), *ν_theor_* 1019, 1031 cm^−1^ for di- (*ν_϶ĸcn_* 1028 cm^−1^), and 1028–993 cm^−1^ (*ν_exp_* 1025 cm^−1^) for tris-hydroxyamidine (THA1).

As a rule, deformation vibrations are observed below 1500 cm^−1^. The vibration frequencies *δ*COH 1114 cm^−1^ (mono-) and 1097 cm^−1^ (tris-hydroxyamidine) [27] correspond to the deformation vibrations of COH and methylene groups in the regions of the mono-, di- and tris-hydroxyamidine theoretical spectra.

#### 2.2.5. Other Vibrations

The vibration of medium intensity is observed at the experimental frequencies *ν_CC_* 1380.23 cm^−1^ (mono-), 1381 cm^−1^ (di-), and 1384.70 cm^−1^ (tris-), which, however, in the theoretical spectra corresponds to the region of mixed deformation vibrations of groups and the stretching vibration of the C-N bond (1385–1299 cm^−1^) (Table 2).

The frequency range of 849 (MHA), 930–820 (DHA) and 932–841 cm^−1^ (THA1) of the calculated spectra of hydroxyamidines corresponds to the stretching vibrations of the N-C(CH_3_) bonds and the deformation vibrations of the CH_2_ groups (*ν_exp_* 924, 871 cm^−1^ (mono-), 936 cm^−1^ (di-), and 937 cm^−1^, 869 cm^−1^ (tris-)). The last vibration *ν_exp_* 800 cm^−1^ is attributed by the authors [27] to the vibration *δ*CNC.

In the calculated spectra, the region of medium frequencies 770 (mono-), 619 (di-) and 734–642 cm^−1^ (tris-) corresponds to C-C stretching vibrations and deformation vibrations of NCN and OH groups. Below 600 cm^−1^, mixed scissor vibrations of methylene groups, symmetric and asymmetric deformation vibrations of methyl groups, and skeletal rocking are observed.

### 2.3. Geometric Architecture of the Associates of Hydroxyamidine with CO_2_

Known mechanisms of the interaction of amines and amidines with CO_2_ involve the formation of carbamates or bicarbonates [1,2,3,4,5,6,7,8,9,10,11,12,13,14,15,16,17,18,19,20,21,22,23,24,25,26,27,28,29,30].

The interaction of hydroxyamidine with CO_2_ is possible in several directions: heteroatoms (nitrogen, oxygen) can be the main reaction centers. As follows from our previous experimental work [27], hydroxyamidines (mono- and tris-), interacting with carbon dioxide, form an ionic liquid (IL), the structure of which was proposed in the form of repeating links RNH^+^…RCOO^−^. The specified ionic associate of the carbonate type corresponds to protonated imino nitrogen =N- and the deprotonated carbonate group, which can be schematically depicted as follows (using the example of monohydroxyamidine):

2R=N(CH_2_)_2_OH + CO_2_ → RN[(CH_2_)_2_]H^+^…ROCOO^−^ (or RNH^+^…RCOO^−^)

In this paper, we tried to model the IL structure in the form of associates of protonated MHA, DHA and THA1 (MHAH^+^, DHAH^+^, THA1H^+^) with MHA-, DHA- and THA1-carbonate (MHACOO^−^, DHACOO^−^ and THA1COO^−^) in different ratios of the initial molecules.

It should be noted that in [27] the capture of carbon dioxide by the oxygen atom of one of the hydroxyl groups of the amidine was assumed. As far as we know, this reaction channel has not been considered by anyone before. Proton transfer is possible in the presence of a proton donor, which can be methanol formed during the synthesis of hydroxyamidine and remaining in the reaction medium. In our recent work [35], methanol was intentionally retained in the system to investigate its influence on CO_2_ fixation. The presence of methanol was confirmed by characteristic signals in the ^1^H and ^13^C NMR spectra.

According to our assumption, the transfer does not occur directly from the carbonate group to the imino nitrogen atom, but by a relay mechanism: first to methanol, then from methanol to the imino nitrogen of the hydroxyamidine. In the present study, methanol is considered within the computational model as an implicit solvent (IEFPCM), to account for its stabilizing hydrogen-bonding effect. No explicit methanol-derived carbonate species were modeled.

**Figure 4:**
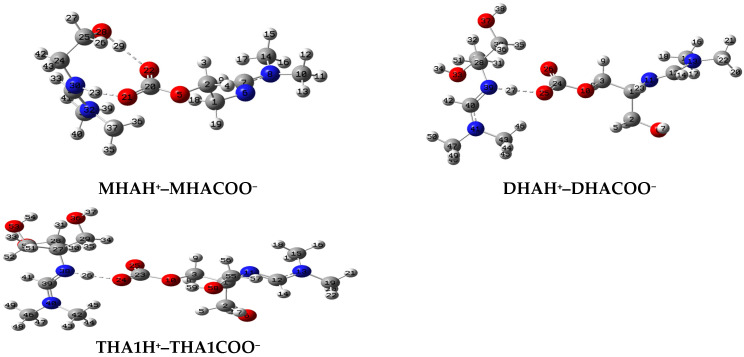
Optimized molecular systems in stoichiometry 2:1 (HA:CO_2_).

**Figure 5:**
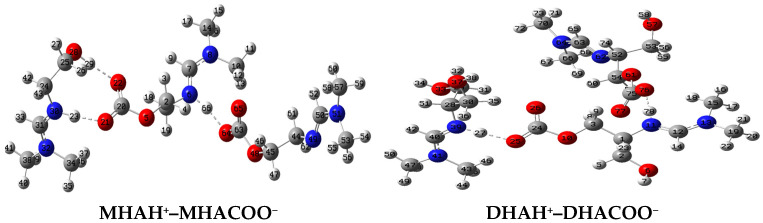
Optimized molecular systems in stoichiometry 3:2 (HA:CO_2_).

**Figure 6:**
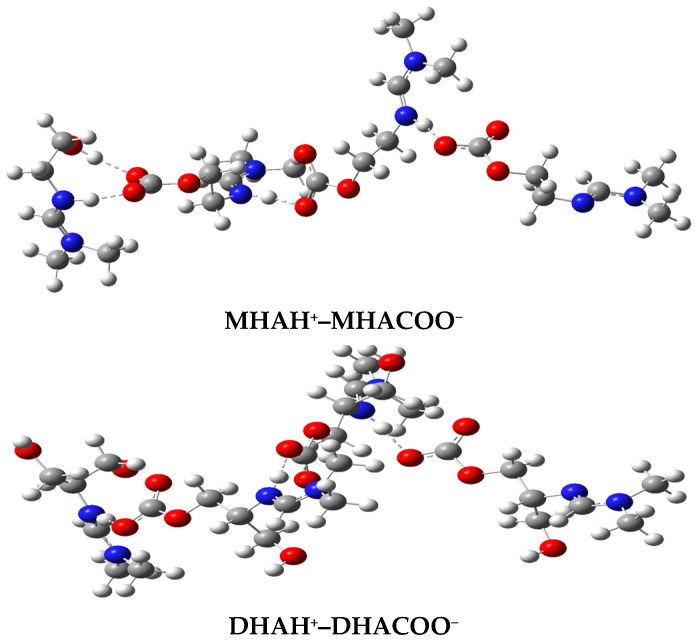
Optimized molecular systems in stoichiometry 4:3 (HA:CO_2_).

The molecular structures of the associates are shown in Figure 4, Figure 5 and Figure 6. Hydrogen bridges are indicated by dotted lines. The internal fragments of the adducts with more than three molecules are zwitterions (Figure 5 and Figure 6). The total energies and binding energies of the associated systems are given in Supplementary Table S2. As the table shows, with an increase in the number of fragments in the associate, the binding energy, which characterizes the strength of the interaction, increases. As expected, this directly depends on the number of hydrogen bonds formed. The energy is negative, which indicates the exothermic nature of the associate formation process.

Not all of the systems studied contain a true H-bond, i.e., an X-H…Y hydrogen bridge. Most of the bridges have the form X…H…Y, where the hydrogen atom is not chemically bonded to any of the electronegative atoms, in our case, to the nitrogen and oxygen atoms: N…H…O. Table 3 shows the internuclear distances and angles between pairs of N…H and H…O atoms, which indicate the linear geometry of the hydrogen bridges (angles are close to 180°) and the presence of one hydrogen bond with an N-H bond length less than 1.06 Å only in the monohydroxyamidine associates (Table 3 and Figure 4, Figure 5 and Figure 6). As expected, all hydrogen bridges are asymmetric; however, the hydrogen atom is shifted to nitrogen (Table 3).

In the absence of a solvent, i.e., in the gas phase, according to electrostatic forces, the proton should shift toward the more electronegative oxygen atom rather than the less electronegative nitrogen atom. In contrast, the presence of methanol provokes interactions between the hydroxyamidine associate and methanol molecules. Such solvent–solute interactions prevail over the electrostatic factor and lead to the displacement of the proton towards the N atom. It should be noted that the distance between the N and O atoms in the systems corresponds to the length of a true H-bond of average strength. There is also a noticeable tendency for the N…O and O…H distances to be shortened and, on the contrary, for r(N…H) to be lengthened with an increase in the number of molecules in the associate (Table 3). In the MHAH^+^–MHACOO^−^ system, an intermolecular hydrogen bond O-H…O is also formed with lengths O-H 0.987 Å, H…O 1.732 Å (Figure 4), O-H 0.988 Å, H…O 1.727 Å (Figure 5), O-H 0.986 Å, H…O 1.742 Å (Figure 6) and angles of 171–172°.

Analysis of the C=N, C=O, and O-C_CO2_ bonds showed that the length of the bond formed between the chemisorbed CO_2_ molecule and the oxygen atom of the hydroxyamidine is in the range of 1.380–1.397 (depending on the amidine type (mono-, di-, tris-), the size of the associate, and the number of sorbed CO_2_), being shorter than a regular O–C bond due to the influence of electron-rich double C=O bonds. An external influence, such as the formation of a hydrogen bond, increases the distance between the atoms, so the C=O double bonds themselves are stretched compared to the bonds in a free carbon dioxide molecule (R_C=O_ = 1.169 Å). In this case, as expected, the C=O bonds involved in the hydrogen bond experience greater stretching (1.262–1.271 Å), while those not involved are less stretched (1.242–1.255 Å). The carbon–nitrogen double bond is also lengthened by 0.03–0.04 Å compared to the original amidine molecules.

**Figure 7:**
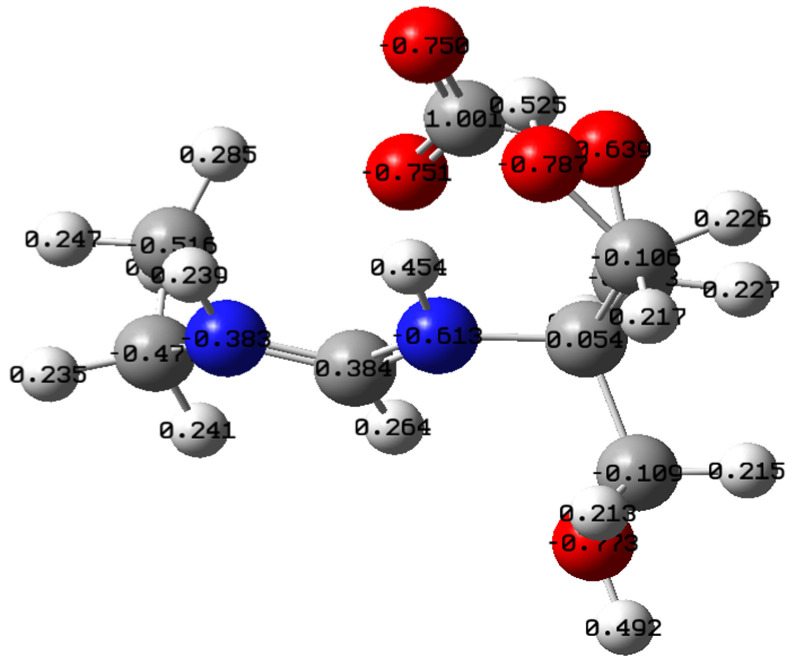
THA2–CO_2_ structure after optimization by B3LYP/6-31G(*d*, *p*) method. E_tot_ = −800.105334 a.u.

The above-considered conformation of tris-amidine THA2 can also absorb carbon dioxide, for example, as follows (Figure 7). The resulting structure resembles a zwitterion (the model is shown with charges on the atoms according to NPA). The geometric parameters of the THA2–CO_2_ system are reported in Supplementary Table S4.

As the calculation results showed, the stretching vibrations of the CO_2_^−^ group in the THA2–CO_2_ system are observed at *ν_calc_* 1709 cm^−1^, and for the C=N bond at 1643 cm^−1^. Unlike the intermolecular associates, there are no additional bands here.

#### 2.3.1. NBO/NPA

Natural population analysis (NPA) allowed us to conclude that the formation of a hydrogen bridge causes a significant redistribution of electron density, polarization of charges on the bridge atoms, and a decrease in density on the nitrogen (the charge becomes more positive), i.e., charge transference between fragments of the associate, leading to the structure of an intermolecular ion pair RNH^+^...RCOO^−^ or RN^+^H…RCOO^−^, which is confirmed by the calculated data in Table 3.

If the isolated fragments have formal −1 and +1 charges, the net charges of each fragment in a hydrogen-bonded associate, for example, MHAH^+^–MHACOO^−^ (2:1) (Figure 4), are, according to NPA, +0.804 and −0.804, respectively (Table 4). A similar charge redistribution is observed for other systems of different stoichiometries (Table 4).

High dipole moments indicate significant delocalization of electron density, as well as the contribution of lone electron pairs. As the number of molecules in the associates increases, the magnitude of the dipole moment increases, but when moving from mono- to di- and tris-hydroxyamidine, the dipole moment decreases, indicating vector compensation.

To assess the degree of electron density delocalization and understand the nature of hydrogen bonding, an NBO analysis of the delocalization energies determined using second-order perturbation theory was performed at the B3LYP/6-31G(*d*, *p*) level of theory. As can be seen from Table 5, the highest delocalization energies are observed for the LP(2)O64 → σ*(N6…H66) and LP(2)O87 → σ*(N49…H89) interactions in monohydroxyamidine associates. These are the most intense interactions with energies of 50 kJ/mol between the electron donor and electron acceptor at monohydroxyamidine to CO_2_ ratios of 3:2 and 4:3.

In the case of dihydroxyamidine, significant electron delocalization, but with lower energy, occurs for the transitions LP(2)O76 → σ*(N11…H78) and LP(2)O76 → σ*(N11…H78). It should be noted that with the appearance of additional hydrogen bonding N…H…O in the systems, subsequent interactions are strengthened. For tris-hydroxyamidine, the electron density is transferred from the lone pair of LP(2)O24 to the adjacent antibonding orbital σ*(H26…N38). Almost twice lower stabilization energies (14–20 kJ/mol) were determined for interactions within O-H…O hydrogen bonds, for example, LP(2)O22 → σ*(O28-H29). All energy values correlate with the H-bonding geometry (Table 3).

#### 2.3.2. Frontier Molecular Orbitals

**Figure 8:**
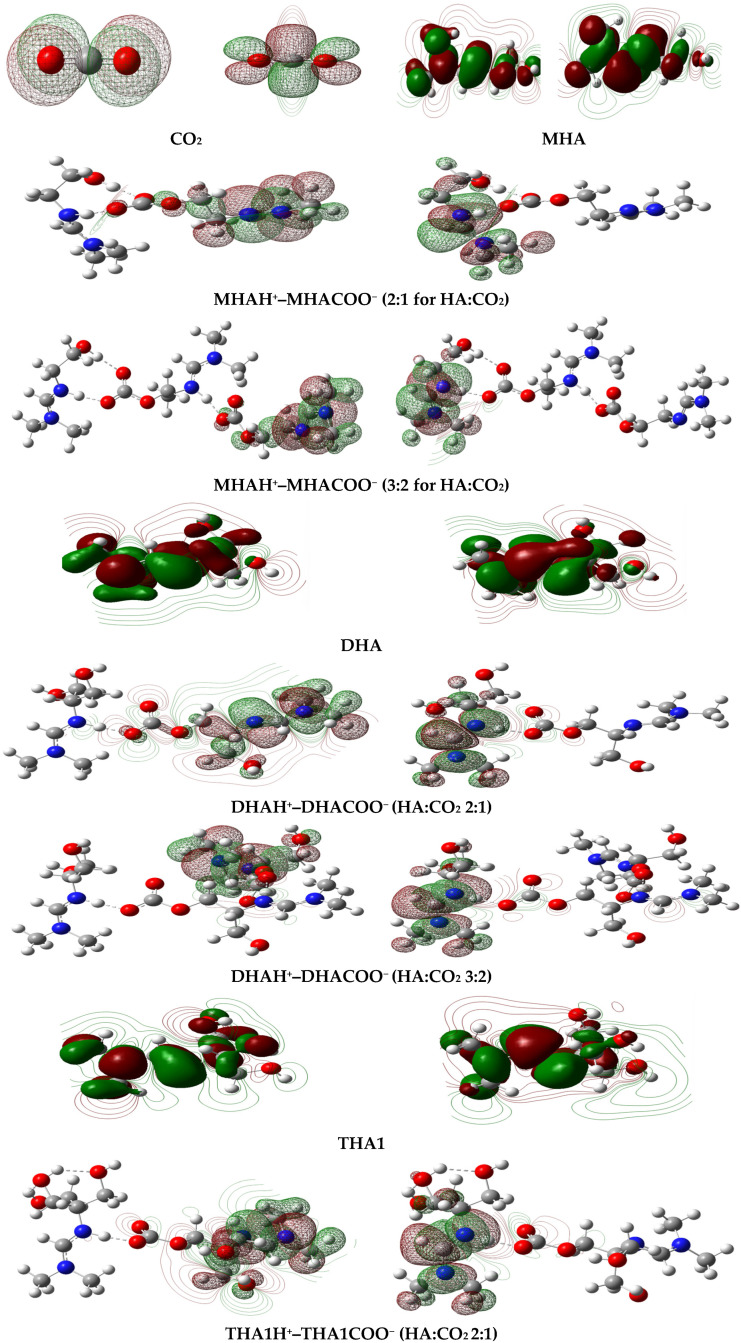
HOMO (**left**) and LUMO (**right**) of isolated molecules and the mono-, di- and tris-hydroxyamidine associates.

It is known that the indicators of electron transfer in molecular systems are the frontier molecular orbitals HOMO (highest occupied molecular orbital) and LUMO (lowest unoccupied molecular orbital). Generated via Gaussian 16, electron density surface plots for these molecular orbitals of isolated molecules and associates are shown in Figure 8. The HOMO of monohydroxyamidine (MHA) is mainly localized on the *p_z_* orbitals of the amino nitrogen, and the LUMO is located on the imino nitrogen atom and the carbon atoms linked to it by a double bond. The HOMO of carbon dioxide is a π-type orbital localized on the oxygen atoms, while the LUMO is π*-characterized and delocalized.

The HOMO and LUMO of the MHAH^+^–MHACOO^−^ complex occupy different regions of the associate, which leads to a significant dipole moment (Table 4). The same is true for di- and tris-hydroxyamidines (Figure 8, Table 4). The calculated energies of the frontier orbitals are given in Supplementary Table S5. The HOMO-LUMO energy gaps of the amidine systems at a HA:CO_2_ ratio of 2:1 are 5.31 eV for MHAH^+^–MHACOO^−^, 5.31 eV for DHAH^+^–DHACOO^−^ and 5.17 eV for THA1H^+^–THA1COO^−^. The smallest energy gap is observed for the tris-hydroxyamidine associate, which favors the formation of the most stable system. A stabilizing factor is the presence of several intra- and intermolecular hydrogen bonds compared to mono- and dihydroxyamidine associates.

#### 2.3.3. Vibrational Analysis of Amidine Systems with CO_2_

As is known, changes in chemical composition and particle association lead to anisotropy of the electron charge distribution in molecular systems and, consequently, to changes in the dipole moments of bonds and molecules, which, in turn, are associated with molecular vibrations.

**Figure 9:**
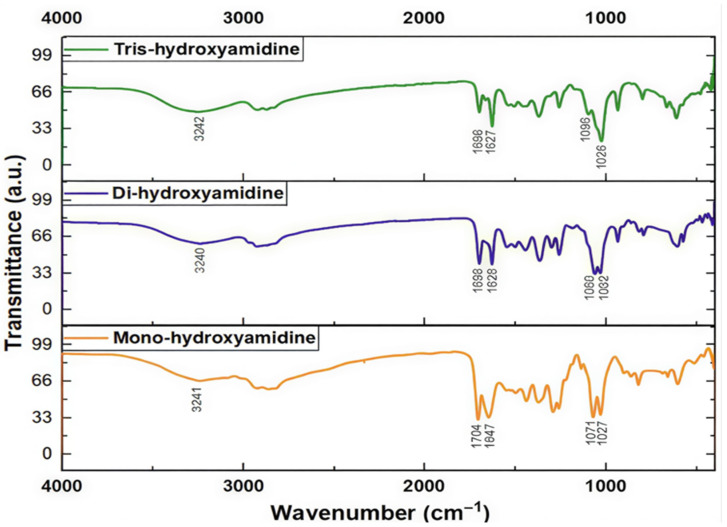
Observed IR spectra of hydroxyamidines treated with CO_2_ gas ([27] and the present work).

**Figure 10:**
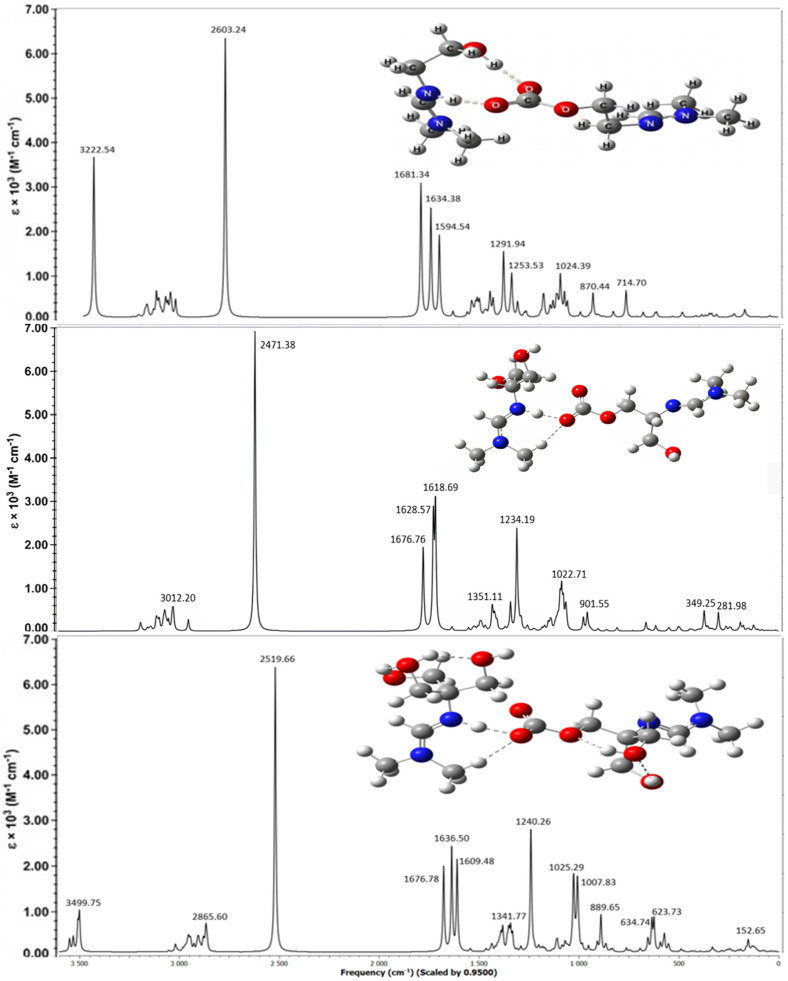
Simulated vibrational spectra of mono-, di-, and tris-hydroxyamidine associates at a H A:CO_2_ ratio of 2:1.

**Figure 11:**
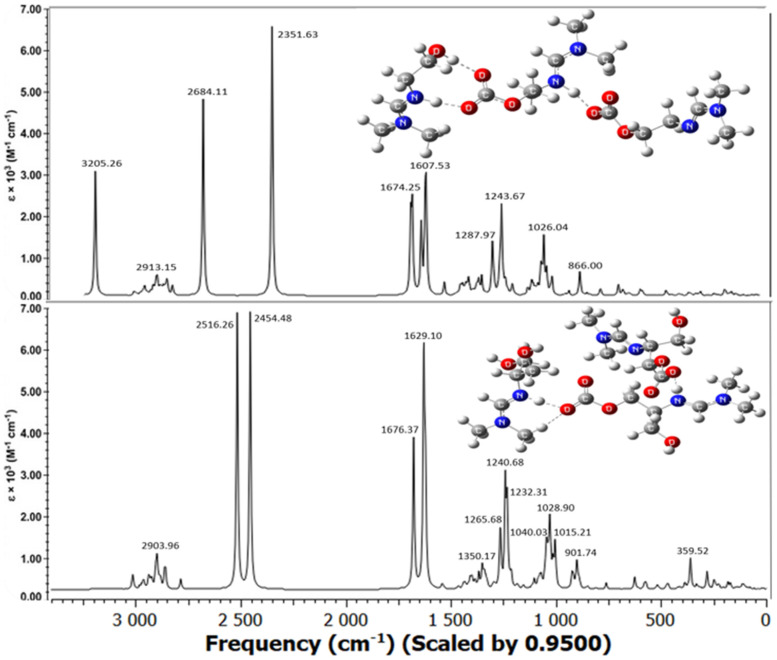
Calculated vibrational spectra of mono-, di-hydroxyamidine associates at a HA:CO_2_ ratio of 3:2.

**Figure 12:**
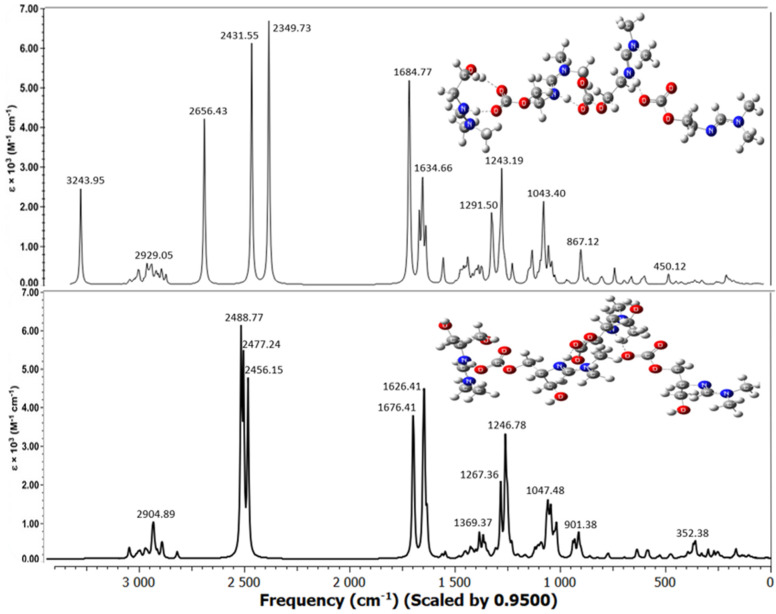
Simulated vibrational spectra of mono-, di-hydroxyamidine associates at a HA:CO_2_ ratio of 4:3.

Below, the experimental IR spectra of mono-, di-, and tris-hydroxyamidines obtained after treating the latter with gaseous carbon dioxide are compared with the theoretically calculated vibrational spectra of amidine systems with CO_2_ (in a methanol medium) (Figure 9, Figure 10, Figure 11 and Figure 12).

The absorption spectra of hydroxyamidine molecules chemically bound to carbon dioxide, calculated by the B3LYP-D3/6-31G(*d*, *p*) method taking into account dispersion effects and using the integral equation formalism (IEF) of the polarizable continuum model (PCM), are shown in Figure 10, Figure 11 and Figure 12. Within the framework of the density functional and basis set used by us, all calculated vibrational spectra are scaled by 0.95.

Figure 10 shows the spectra of mono-, di-, and tris-hydroxyl-containing amidines at a HA:CO_2_ ratio of 2:1, while Figure 11 and Figure 12 show the spectra of mono- and dihydroxyl-containing amidines at HA:CO_2_ ratios of 3:2 and 4:3, respectively. A comparison of experimental and calculated frequencies for hydroxyamidine-carbon dioxide systems showed satisfactory agreement between them. Scaled values of vibrational frequencies and IR band intensities are presented in Table 6.

As noted above, the lack of signals in the region > 3550 cm^−1^ in the experimental spectra indicates that there are no free OH groups in the IL, and all hydroxyl groups of the MHA, DHA, and THA ILs participate in the formation of hydrogen bonds, both intra- and/or intermolecular ones. An intense broad band is observed in the region of 3240–3242 cm^−1^ in the experimental spectra of mono-, di-, and tris-hydroxyamidines, which corresponds to the intermolecular O-H…O hydrogen bond formed between the hydroxyl group of the amidine molecule and the COO^−^ carbonate group. The theoretical frequency of 3244 cm^−1^ for the tris-hydroxyamidine associate is closest to these values. The absorption bands *ν_ex_*_p_ 2859 cm^−1^, 2928 cm^−1^ and 2927 cm^−1^ of the MHA, DHA and THA ILs, respectively, belong to the stretching vibrations of the C–H bonds of the methylene and/or methyl groups ([27] and the present work). In the theoretical spectra of the systems, the absorption region in the range of 3012–2860 cm^−1^ also corresponds to the asymmetric and symmetric vibrations of the C–H bonds of the indicated groups (Table 6).

Some of the most intense bands in the experimental IR spectra of the IL MHA (*ν_exp_* 1704 and 1647 cm^−1^), DHA (*ν_exp_* 1698 and 1628 cm^−1^) and THA (*ν_exp_* 1698 and 1627 cm^−1^) are due to the stretching vibrations of double bonds: C=O and C=N, respectively. In the theoretical spectra, these vibrations appear at 1595, 1677, 1677 (C=O) and 1634, 1629, 1637 (C=N) cm^−1^ for the MHA, DHA, and THA1 associates, respectively.

It should be noted that our systems simulate the binding of the oxygen of the carbonate group to the hydrogen located near the imino nitrogen. The formation of hydrogen bonding weakens and lengthens the double bonds, and changes the frequency of their vibrations; therefore, the absorption bands are shifted to the low-frequency region.

The calculated spectra also contain a very intense band corresponding to the N^+^…H vibration (ν_theor_ 2350–2603 cm^−1^ in the MHA, DHA, and THA1 associates). This can be compared, for example, to the IR spectrum of the zwitterionic form of the amino acid glycine, where N^+^–H stretching vibrations are observed in the region above 2000 cm^−1^ [36]. However, the experimental spectra of mono-, di-, and tris-hydroxyamidines do not exhibit a similar band. We assume that the N^+^–H stretching vibrations (2350–2600 cm^−1^) do not appear in the experimental spectra because the bond between the nitrogen atom and the proton is not a true chemical bond, i.e., the N^+^–H bond. As we noted above, the proton is indeed shifted toward the imino nitrogen atom and is quite close (1.05–1.06 Å), but is practically not chemically bound.

Mixed vibrations of single and double carbon–nitrogen bonds are observed in the region of *ν_theor_* 1609–1681 cm^−1^, and those of carbonate C-O bonds are observed in the region of *ν_theor_* 1232–1292 cm^−1^ in mono-, di-, and tris-hydroxyamidine associates. Vibration frequencies below 1050 cm^−1^ of the theoretical spectra characterize deformation vibrations of OH and methylene groups, and changes in the valence angles *δ*(OCO), *δ*(HCH), *δ*(NCN), *δ*(COH).

Previously [27] we observed the appearance of a new band between 1700 and 1500 cm^−1^ in the spectra of mono- and tris-hydroxyamidines treated with CO_2_ and attributed this vibration to the formation of the amidinium group C=N^+^. A similar band was also recorded in the spectrum of di-hydroxyamidine (this work, Figure 9). The results of vibrational calculations are consistent with these facts: additional bands in the indicated region appear in the spectra of the MHA, DHA, and THA associates (Figure 10, Figure 11 and Figure 12, Table 6).

#### 2.3.4. Comparison of Chemical Shifts in the Spectra of the Tris-Hydroxyamidine Associate

^1^H NMR spectra of some tris-hydroxyamidine systems were also calculated in the work. Table 7 presents the experimental and theoretical (B3LYP/6-311+G(2*d*, *p*) method) chemical shifts of ^1^H nuclei for tris-hydroxyamidine, ionic liquid and model systems. The signals of protons at the CN double bond in the calculated spectra of tris-hydroxyamidine and associates are strongly shifted to a weak field compared to the experimental spectra of tris-hydroxyamidine and IL.

Downfield shifts are observed for the signal of OH-group protons in methanol molecules. On the contrary, an upfield shift is manifested for the signal of OH-group protons of hydroxyamidine molecules. In the experimental ^1^H NMR spectrum of IL [27], an assignment was made for the chemical shift of 5.20 ppm as belonging to the ^1^H nucleus of the intermolecular hydrogen bond between the oxygen atoms of the CO_2_ group and the iminonitrogen of the hydroxyamidine. In the calculated systems with CO_2_, the corresponding signals are extremely strongly shifted to a weak field.

Calculations of amidine molecule systems with bridging CO_2_ groups in methanol improved the results for the H-bond proton: they showed an upfield shift in the aforementioned signal compared to gas-phase calculations, which better matches the experimental results.

## 3. Materials and Methods

### 3.1. Synthesis Method for Di-Hydroxyamidine

Chemical Reagents: The following reagents were used for the synthesis of amidine: 2-amino-2-methyl-1,3-propanediol (C_4_H_11_NO_2_, M = 105.14 g/mol, purity ≥ 99%, Sigma-Aldrich, St. Louis, MO, USA) and N,N-dimethylformamide dimethyl acetal (C_5_H_13_NO_2_, M = 119.16 g/mol, purity ≥ 97%, Thermo Fisher Scientific, Waltham, MA, USA). All reagents were used without further purification.

General information on the synthesis of amidine based on hydroxy-containing amine: amidine with hydroxyl substituents is obtained by reacting the corresponding amino alcohol with N,N-dimethylformamide dimethyl acetal (DFDA). The process is carried out at a 1:1 molar ratio of the reagents using a magnetic stirrer. The reaction is carried out in two stages: initial stirring at 40–50 °C until the starting amino alcohol is completely dissolved (3 h), then raising the temperature to 60 °C, followed by additional stirring for 30 min. Next, the amidine is treated with CO_2_ gas.

Synthesis of dihydroxyamidine:

2-Amino-2-methyl-1,3-propanediol (5.26 g, 0.05 mol) was gradually (0.3–0.4 g at a time) added to a solution of dimethylformamide dimethyl acetal (6.90 mL, 0.05 mol). After complete dissolution of the reagent, the mixture was maintained under standard synthesis conditions. The target product was isolated in 97% yield.

The synthesis method for mono- and tris-hydroxyamidines was described in [27].

Fourier transform infrared spectroscopy (FTIR) was employed to examine the characteristic functional groups of dihydroxyamidine and PIL. Spectra were recorded using a Nicolet™ iS10 FTIR spectrometer (Thermo Scientific, Waltham, MA, USA) fitted with a diamond attenuated total reflectance (ATR) accessory. Each sample was analyzed directly in ATR mode without additional preparation. The spectra were acquired in the range of 4000–500 cm^−1^, with a spectral resolution of 4 cm^−1^, and 32 individual scans were averaged for each run to improve the signal-to-noise ratio. The raw data was processed and interpreted using Omnic 5.2 and OriginPro-2024 softwares. Spectra of mono- (FTIR) and tris-hydroxyamidines (FTIR, ^1^H NMR and ^13^C NMR recorded in CDCl_3_) from our work [27] were used for comparative analysis.

FTIR and NMR analysis coupled with theoretical modeling.

### 3.2. Computational Details

Quantum chemical calculations of molecular structure and properties of the studied systems were carried out by the density functional theory (DFT) using the closed-shell three-parameter hybrid exchange-correlation functional Becke 3 Lee Yang Parr (B3LYP). The valence-split 6-31G(*d*, *p*) basis set augmented by *d* polarization functions on heavy atoms and *p* polarization functions on hydrogen atoms [37].

Full geometry optimization was performed for all systems under consideration without imposing symmetry constraints, regardless of the local symmetry of the alkyl groups comprising the systems. The environment was accounted for at the B3LYP/6-31G(*d*, *p*) level using the intrinsically independent polarizable continuum model (IEFPCM). As expected, the addition of methanol as a medium lowered the overall energy of the systems. The DFT-D3 method [38] was used to account for dispersion effects during complex formation.

Vibrational frequencies were computed for the optimized structures using the same method in order to assess the nature of stationary points and to obtain spectroscopic characteristics. The resulting structures were identified as stationary points (minima) on the potential energy surface (all vibrational frequencies of the optimized structures are valid). To ensure consistency between the calculated spectra and the experimental ones, a scaling factor of 0.95 was applied, which is within the range of expected values for the density functional and basis set used in our calculations [34].

Natural population analysis (NPA), based on NBO (Natural Bond Orbital) analysis [39,40], was used for a comparative study of the charge distribution over atoms in isolated components and complexes. NPA was chosen for the following reasons: it provides a better description of the electron distribution, especially for systems with an ionic or polar character. NBO analysis was also performed to determine orbital contributions to charge transfer between fragments of the complexes. The electronic state of all systems corresponds to the ground singlet state.

NMR spectra of tris-hydroxyamidine and its associates were calculated at DFT level employing B3LYP/6-311+G(2*d*, *p*). All calculations were performed using the Gaussian16 software package [37]. The visualizations of the calculated spectra were **done** using the GaussView 6.0.16, Chemcraft 1.8, Multiwfn 3.8 programs [41,42,43].

## 4. Conclusions

Structural, electron-energetic, and spectral data from DFT calculations for mono-, di-, and tris-hydroxyamidines and their associates with CO_2_ are presented. Optimal minimum-energy conformations of the molecules are identified. Another possible conformation with the probability of forming an intramolecular O-H…N hydrogen bond is discussed for tris-hydroxyamidine. Experimental and theoretical IR absorption spectra correlate with the structure of hydroxyamidines and are consistent with each other. The previously proposed structure of ionic liquids in the form of repeating RNH^+^…RCOO^-^ units is modeled in this work as associates of protonated mono-, di-, and tris-hydroxyamidines with carbonate counterions in different ratios of the initial molecules. The formation of these units is facilitated by nontrivial sites of CO_2_ absorption and hydroxyamidine protonation, namely, the oxygen atom of one of the hydroxyl groups of the amidine (CO_2_ chemisorption) and the imino nitrogen as a proton acceptor. The internal fragments of the associates for molecules greater than three are zwitterions. The present DFT results are in fairly good agreement with the experimental data: they confirm the appearance of a new band between 1700 and 1500 cm^−1^ in the spectra of model PILs of mono-, di-, and tris-hydroxyamidenes, previously attributed to the formation of the amidinium group C=N^+^, and support new insight into the formation of the PIL structure.

## Figures and Tables

**Table 1:** Total electronic energies of amidine molecules (B3LYP/6-31G(*d*, *p*) method).

Molecule	−E_t_, a.u.	E_0_, a.u.	−(E_t_ + E_o_), a.u.
MHA	382.472334	0.175994	382.296340
DHA	497.034146	0.209152	496.824994
THA1	611.535204	0.243583	611.291621
THA2	611.530028	0.243116	611.286912

**Table 2:** Observed and calculated (B3LYP/6-31G(*d*, *p*) method) scaled frequencies (*ν*) and intensities (*I*) of stretching and mid-frequency vibrations in mono-, di- and tris-hydroxyamidine molecules.

Vibration Type	Monohydroxyamidine			Dihydroxyamidine			Tris-Hydroxyamidine		
	Calc. (ν), cm^–1^	Exp. [27] (ν), cm^–1^	*I* (calc.)	Calc. (ν), cm^–1^	Exp. [This Work] (ν), cm^–1^	*I* (calc.)	Calc. (ν), cm^–1^	Exp. [27] (ν), cm^–1^	*I* (calc.)
*ν*(O-H) *	3619	3294	13	3643 3619	3319	18 11	3650 3555 3544 (3471) **	3309	31 77 109 144
*ν_as_*_,_*_s_*(CH_3_, CH_2_, CH) (out of phase, in phase)	2970 2941 2927 2913 2868 2862 2856 2853 2843 2809	2912 2827	22 45 35 42 46 76 89 49 74 75	2972 2951 2933 2918 2898 2888 2881 2858 2845 2786	2930	24 31 38 44 45 60 53 85 71 57	2975 2955 2938 2934 2921 2909 2881 2863 2858 (2856) 2848	2936 2828	16 30 34 8 36 52 42 53 128 113 69
*ν*(C=N)	1655	1645	515	1655	1629	562	1623 (1630)	1629 (53) ***	573 569
*δ*(CH_3_), *δ*(CH_2_)	1471–1407	1438 1406	8 14	1470–1408	1438	8 16	1472–1406	-	7 24
*ν*(C-N), *ρ*(CH), *δ*(OH), *δ*(CH_3_), *w*(CH_2_), *τ*(CH_2_), *δ*(COH)	1374 1337 1325 1266 1226	1380 1345 (76) 1264 (78) 1114 (69)	30 62 78 28 38	1374 1344 1338 1292 1232 1184 1118 1094	1381 1259	17 22 83 27 34 43 23 34	1380 1361 1347 (1350) 1338 1299 1231 1166 1101	1384 (76) 1096 (66)	106 64 117 107 19 6 24 19 77
*ν*(C(CH_3_)-N), *δ*(CCC), *ρ*(CH), skeleton swing	1075	1063 (66)	76	1063 1041 1037	1096	73 9 10	1065 1049 (1059) 1036	-	31 69 127 6
*ν_as_*_,_*_s_*(C-O), skeleton swing	1025	1030 (41)	162	1031 1019 1004	1028	101 83 59	1028 1013 993 (992)	1025 (38)	145 118 92 103
*ν_s_*(C-N), *δ*(HCH), *ρ*(CH_2_), *τ*(CH), *δ*(OH), skeleton swing	984 937 849	924 (83) 871 (78)	18 8	930 889 858 820	936	2 22 3 4	983 932 859 841	937 (71) 869 (78)	6 13 5 14
*ρ*(OH), *ν_s_*(C-C), *ρ*(OH), *δ*(NCN), skeleton swing	770	-	2	619 518	794 630 573	4 22	734 660 642 (680)	800 (74)	4 77 152 143

* *ν*—stretch vibration, *ν_as_*—asymmetric stretch vibration, *ν_s_*—symmetric stretch vibration, *ν*(C-C)—stretch vibrations of the carbon skeleton; deformation vibrations: *δ*—change in the valence angle; *ρ*—pendulum; *w*—fan; *τ*—torsional. ** Vibrational mode in THA2. *** Vibration intensity in the experimental spectrum.

**Table 3:** Hydrogen bond geometry (B3LYP/6-31G(*d*, *p*) method) in MHA, DHA, and THA1 associates (Figure 4, Figure 5 and Figure 6).

Parameter	r(N…O), Å	r(N…H), Å	r(O…H), Å	φ(N… H…O), °
HA:CO_2_	MHAH^+^–MHACOO^−^			
2:1	2.663 ^a^	1.061	1.614	168.94
3:2	2.702	1.055	1.648	175.88
	(2.619) ^b^	(1.079)	(1.556)	(167.02)
4:3	2.674	1.057	1.630	168.46
	(2.619	(1.073	(1.562	(166.98
	2.598)	1.079)	1.539)	165.50)
	DHAH^+^–DHACOO^−^			
2:1	2.662	1.069	1.594	176.85
3:2	2.669	1.066	1.604	176.43
	(2.659)	(1.070)	(1.590)	(175.82)
4:3	2.646	1.068	1.580	176.59
	(2.646	(1.068	(1.579	(175.26
	2.636)	1.071)	1.581)	167.01)
	THA1H^+^–THA1COO^−^			
2:1	2.654	1.065	1.592	174.33

^a^ Internuclear distances N…O, N…H, and O…H, and the N…H…O angle for the first hydrogen bridge in the associates. ^b^ Internuclear distances and angle for other hydrogen bridges in the associates.

**Table 4:** Electronic charges of hydrogen bond atoms (B3LYP/6-31G(*d*, *p*), NBO method) in mono-, di- and tris-hydroxyamidine associates.

	Natural Atomic Charge					µ, D
	N	H	O	HAH^+^	HACOO^−^	
HA:CO_2_	MHAH^+^–MHACOO^−^					
-	−0.582	0.447	−0.774	+1	−1	2.463 ^a^ (16.866)
2:1	−0.581	0.466	−0.773	0.804	−0.804	8.918
3:2	−0.570 (−0.570) ^b^	0.470 (0.475)	−0.786 (−0.788)	0.838	−0.838	15.163
4:3	−0.580 (−0.569, −0.568)	0.466 (0.474, 0.474)	−0.766 (−0.774, −0.792)	0.841	−0.841	23.429
	DHAH^+^–DHACOO^−^					
-	−0.584	0.455	−0.807	+1	−1	5.878 (21.229)
2:1	−0.578	0.473	−0.809	0.836	−0.836	8.497
3:2	−0.578 (−0.571)	0.473 (0.473)	−0.808 (−0.807)	0.837	−0.837	11.963
4:3	−0.580 (−0.574, −0.574)	0.471 (0.471, 0.472)	−0.802 (−0.800, −0.786)	0.839	−0.839	12.639
	THA1H^+^–THA1COO^−^					
-	−0.592	0.456	−0.785	+1	−1	7.981 (17.558)
2:1	−0.588	0.470	−0.799	0.845	−0.845	6.418

^a^ Dipole moments of the HAH^+^ and HACOO^−^ systems, respectively. ^b^ Charges on analogous atoms.

**Table 5:** Delocalization energies of mono-, di-, and tris-hydroxyamidine associates at the B3LYP/6-31G(*d*, *p*) level of theory.

HA:CO_2_	Delocalization	Energy, kJ/mol		
		MHAH^+^– MHACOO^−^	DHAH^+^– DHACOO^−^	THA1H^+^– THA1COO^−^
2:1	LP(2)O21 → σ*(H23…N30)	37.52	-	-
	LP(2)O22 → σ*(O28-H29)	15.44	-	-
	LP(2)O25 → σ*(H27…N39)	-	42.19	-
	LP(2)O24 → σ*(H26…N38)	-	-	42.45
3:2	LP(2)O21 → σ*(H23-N30)	33.08	-	-
	LP(2)O22 → σ*(O28-H29)	19.57	-	-
	LP(2)O64 → σ*(N6…H66)	49.87	-	-
	LP(2)O25 → σ*(H27…N39)	-	40.04	-
	LP(2)O76 → σ*(N11…H78)	-	44.23	-
4:3	LP(2)O21 → σ*(H23-N30)	35.74	-	-
	LP(2)O22 → σ*(O28-H29)	14.24	-	-
	LP(2)O64 → σ*(N6…H66)	47.96	-	-
	LP(2)O87 → σ*(N49…H89)	49.80	-	-
	LP(2)O25 → σ*(H27…N39)	-	41.08	-
	LP(2)O76 → σ*(N11…H78)	-	45.10	-
	LP(2)O103 → σ*(N62…H105)	-	42.08	-

**Table 6:** Experimental and theoretical (B3LYP/6-31G(*d*, *p*) method) scaled frequencies (ν) and intensities (I) of stretching and mid-frequency vibrations in the MHA, DHA, and THA systems with CO_2_ at different HA:CO_2_ ratios.

Type of Vibrations	MHA-CO_2_			DHA-CO_2_			THA1-CO_2_		
	Calc. (ν), cm^–1^	Exp. [27] (ν), cm^–1^	*I* (calc.), km/mol	Calc. (ν), cm^–1^	Exp. [Present Work] (ν), cm^–1^	*I* (calc.), km/mol	Calc. (ν), cm^–1^	Exp. [27] (ν), cm^–1^	*I* (calc.), km/mol
*ν*(O-H) *	3223 (3205 **, 3244)	3241	1114 1345 1072	-	3240	-	3500	3242	323
*ν_as_*_,*s*_(CH_3_, CH_2_) (out of phase, in phase)	2862 (2861, 2929)	2859	121 129 100	2862 (2861, 2905)	2928	149 151 72	2866	2927	144
*ν*(N…H)	2603 (2352, 2350)	-	1941 2903 2944	2471 (2454, 2477)	-	2726 2694 2497	2520	-	2487
*ν_as_*(CO_2_^−^)	1595 (1608, 1621)	1704	565 1014 721	1619 (1621, 1626)	1698	1087 1028 1180	1677	1698	741
*ν*(C=N), *ν*(C-N)	1681 (1674, 1685)	1647	930 916 1155	1629 (1629, 1676)	1628	981 1021 1034	1637 1609	1627	899 789
Mid frequencies									
*δ*(COH), *τ*(CH_2_), *δ*(HCH)	1416 (1415, 1415)	1497 1439	97 90 104	1541 (1542, 1529)	1544 1500 1444	36 38 90	1544 1437	1505 1440	27 27
*ρ*(CH), *w*(CH_2_)	1355 (1354, 1354)	1370	159 159 165	1351 (1350, 1369)	1364	155 172 271	1342	1371	184
*ν*(C(O_2_)-O), *w*(CH_2_), skeleton swing	1292 1254 (1288 1244, 1292 1243)	1290 1259	453 284 545 919 647 1182	1264 1234 (1241 1232, 1247 1236)	1298 1258	248 921 994 784 1443 652	1240	1258	1022
*ν*(C(H_2_)-O), *δ*(COH), *ρ*(CH_2_), skeleton swing	1024 (1040, 1043)	1137 1071	276 574 595	1023 (1029, 1047)	1060 1032	330 540 290	1025 1007	1096 1026	429 514
*δ*(OCO), *ρ*(CH_2_), *δ*(HCH), *δ*(NCN), skeleton swing	870 (866, 867)	865 822	169 212 247	902 (902, 901)	935 793	163 191 173	890	935 799	322

* *ν*—stretch vibration, *ν_as_*—asymmetric stretch vibration, *ν_s_*—symmetric stretch vibration, *ν*(C-C)—stretch vibrations of the carbon skeleton; deformation vibrations: *δ*—change in valence angle; *ρ*—pendulum vibration; *w*—fan vibration; *τ*—torsional vibration. ** Frequencies at other HA:CO_2_ ratios.

**Table 7:** Experimental and calculated (B3LYP/6-311+G(2*d*, *p*) method) chemical shifts of ^1^H nuclei in some tris-hydroxyamidine systems.

System	Chemical Shift δ, ppm									
	^1^H(CH_3_)		^1^H(CH_2_)		^1^H(OH)		^1^H(C=N)		^1^H(N)	
	Exp. [27]	Calc.	Exp. [27]	Calc.	Exp. [27]	Exp.	Exp. [27]	Calc.	Exp. [27]	Calc.
THA1	3.60	2.01	3.07	3.45	1.26	0.31	6.96	8.81	-	-
	3.62	2.06	4.19	3.58		2.66				
	3.67	2.72	4.20	3.67		3.14				
	3.70	2.73		4.02						
	3.73	3.07		4.04						
		4.23		4.82						
PIL	3.28	-	3.48	-	4.06	-	6.86	-	5.20	-
THA1H^+^- THA1COO^−^		1.92		3.01		2.64		8.45		15.11
		2.12		3.26		3.03		8.75		
		2.34		3.43		3.77				
		2.49		3.49		5.90				
		2.70		3.52		6.47				
		2.81		3.71						
		3.00		3.80						
		3.01		4.29						
		3.017		4.53						
		3.02		4.71						
		4.31		5.05						
		5.51		5.13						
THA1H^+^- THA1COO^−^ with implicit methanol solvation		2.18		3.12		2.67		8.28		11.37
		2.33		3.38		2.77		8.70		
		2.63		3.51		3.77				
		2.79		3.71		6.09				
		2.82		3.729		6.92				
		2.96		3.731						
		3.04		3.82						
		3.18		4.15						
		3.22		4.50						
		3.27		4.64						
		3.75		4.66						
		4.53		4.94						

## Data Availability

The original contributions presented in the study are included in the article/Supplementary Material; further inquiries can be directed to the corresponding author.

## Supplementary Materials

The following supporting information can be downloaded at: https://www.mdpi.com/article/10.3390/molecules31061055/s1, Table S1: The structural parameters for the equilibrium geometry of hydroxyamidines; Table S2: The total and binding energies of the hydroxyamidine associates; Table S3: The key structural parameters of hydroxyamidine associates; Table S4: The theoretical values of geometric parameters for the THA2–CO_2_ system; Table S5: The calculated values of the HOMO-LUMO energies for the systems studied.
