# Supplementary material for: Molecular Structure, Theoretical NBO Analysis, Vibrational Spectrum of CO2-Responsive Hydroxyamidine-Based Ionic Liquid: A Combined Computational and Experimental Approach
Source: Molecules. 2026 Mar 23;31(6):1055. doi: 10.3390/molecules31061055 (PMC13029044; doi:10.3390/molecules31061055)
Supplement: Supplementary file 1 [file molecules-31-01055-s001.zip › molecules-4170521-supplementary.pdf]

SUPPLEMENTARY MATERIALS

Molecular Structure, Theoretical NBO Analysis, Vibrational Spectrum of CO_2-Responsive Hydroxyamidine-Based Ionic Liquid: A Combined Computational and Experimental Approach

Lyazzat Abulyaissova [1,*], Nikolay Barashkov [2], Irina Irgibaeva [3] and Yerbolat Tashenov [3]

[1] Chemistry Department, Buketov National Research University, Karaganda 100028, Kazakhstan
[2] Micro-Tracers, Inc., 1370 Van Dyke Avenue, San Francisco, CA 94124, USA
[3] Department of Chemistry, L.N. Gumilyov Eurasian National University, Astana 010008, Kazakhstan
* Correspondence: abu.lyazzat@gmail.com

Content

Table S1. The structural parameters for the equilibrium geometry of hydroxyamidines.

Table S2. The total and binding energies of the hydroxyamidine associates.

Table S3. The key structural parameters of hydroxyamidine associates.

Table S4. The theoretical values of geometric parameters for the THA2 - CO_2 system.

Table S5. The calculated values of the HOMO-LUMO energies for the systems studied.

All the numerical data were obtained using the method B3LYP/6-31G(*d*, *p*) method.

Table S1. The structural parameters for the equilibrium geometry of hydroxyamidines.

**Parameter**	**Monohydroxyamidine**	**Dihydroxyamidine**	**Tris-hydroxyamidine**	
	MHA	DHA	THA1	THA2
Bond length (Å)				
C=N	1,279	1,278	1,285	1,284
C(H)-N	1,371	1,370	1,369	1,368
N-C_{CH3}	1,452<br>(1,449)	1,451<br>(1,448)	1,451<br>(1,448)	1,450<br>(1,452)
N-C(CH_2)	1,449	1,449	1,465	1,464
C-C	1,534	1,536 (1,527)	1,539<br>(1,551,<br>1,554)	1,542<br>(1,547,<br>1,552)
C-O	1,423	1,4249<br>(1,4248)	1,423<br>(1,426,<br>1,436)	1,420<br>(1,433,<br>1,416)
O-H	0,966	0,966 (0,965)	0,964<br>(0,971,<br>0,972)	0,965<br>(0,972,<br>0,975)
C(O)-H	1,095<br>(1,100)	1,100 (1,101)<br>1,094 (1,097)	1,097 (1,100)<br>1,093 (1,100)<br>1,095 (1,098)	1,101(1,103)<br>1,094(1,095)<br>1,098(1,103)
C_{CH3}-H	1,093<br>(1,096,<br>1,101)	1,093<br>(1,096,<br>1,101)	1,093<br>(1,097,<br>1,101)	1,093<br>(1,096,<br>1,100)
C(=N)-H	1,101	1,097	1,095	1,093
Bond angle (º)				
N=C-N	123,846	123,407	122,884	122,398
C=N-C	117,163	116,923	118,109	120,968
C-N-C	120,213<br>(119,584)	119,693<br>(118,937)	120,606	120,346<br>(119,972)
N-C-C	110,729	110,212<br>(108,284)	104,556<br>(109,045,<br>115,144)	105,111<br>(109,373,<br>116,118)
C-C-O	112,449	112,116<br>(108,335)	109,209<br>(113,798,<br>113,128)	110,117<br>(110,571,<br>113,836)
C-O-H	107,454	107,966<br>(106,907)	108,719<br>(105,268,<br>104,568)	107,711<br>(105,775,<br>103,917)
Dihedral angle (º)				
C-N-C=N	-164,527	-163,392	-168,668	-164,916
N-C=N-C	-174,912	-179,741	-176,188	-173,628
C=N-C-C	116,546	148,675<br>(-89,488)	-162,364<br>(80,202,<br>-43,217)	-134,262<br>(108,130,<br>-13,236)

N-C-C-O	176,802	-175,333 (68,008)	-170,521 (-168,880, 83,548)	-54,228 (61,438, 57,476)

Table S2. The total and binding energies of the hydroxyamidine associates.

	E_{tot} (a.u.)	ΔE_{bind} (kcal/mol)
**HA:CO_2**	**$MHAH^+$ - $MHACOO^-$**	
2:1	-953,653569	-192,347
3:2	-1524,678072	-218,987
4:3	-2095,920901	-381,193
	**$DHAH^+$ - $DHACOO^-$**	
2:1	-1182,636132	-142,085
3:2	-1868,260720	-216,253
4:3	-2554,067238	-404,400
	**$THA1H^+$ - $THA1COO^-$**	
2:1	-1411,808925	-200,397

The binding energy is found using the following formula:

$E_{bind} = E_{ass} - \sum E_{frag}$,

E_{bind} is a binding energy, E_{ass} is the associate energy, $\sum E_{frag}$ is a sum of the molecular fragment energies.

Table S3. The key structural parameters of hydroxyamidine associates.

Parameter	Monohydroxyamidine			Dihydroxyamidine			Tris-hydroxyamidine
Bond length (Å)	2MHA	3MHA	4MHA	2DHA	3DHA	4DHA	THA1
C=N	1,313	1,311; 1,312	1,313; 1,310; 1,309	1,311	1,313; 1,312	1,311; 1,311; 1,311	1,315
C=O	1,264 (1,255)	1,269; 1,262 (1,246; 1,252)	1,261; 1,265; 1,269 (1,255; 1,246; 1,244)	1,271 (1,243)	1,269; 1,271 (1,242; 1,242)	1,269; 1,269; 1,268 (1,242; 1,241; 1,246)	1,270 (1,239)
O-C_{CO2}	1,380	1,393; 1,388	1,385; 1,396; 1,391	1,388		1,393; 1,394; 1,388	1,397

Table S4. The theoretical values of geometric parameters for the THA2 - CO_2 system.

Bond length	Value (Å)	Bond angle	Value (º)	Dihedral angle	Value (º)
C=N	1,320	N=C-N	124,300	C-N-C=N	11,760 (165,046)
C(H)-N	1,322	C=N-C	124,136	N-C=N-C	-163,380
N-C_{CH3}	1,471 (1,466)	C-N-C	119,967 (118,032)	C=N-C-C	-77,327 (47,309, 164,034)
N-C(CH_2)	1,485	N-C-C	110,626 (104,063, 110,663)	N-C-C-O	66,994 (-28,292, 67,483)
C-C	1,571 (1,527, 1,555)	C-C-O	112,814 (108,510, 109,664)	C-O-C=O	130,168 (-46,388)
C-O	1,410 (1,425, 1,406)	C-O-H	110,379 (108,582)	C-C-O-H	-179,025 (-62,574)
O-H	0,992 (0,965)	C-O=C	113,707	-	-
C(O)-H	1,096 (1,097) 1,101 (1,100) 1,096 (1,094)	O-C=O	111,203 (116,489)	-	-
C_{CH3}-H	1,094 (1,090, 1,097)	O=C=O	132,192	-	-
C(=N)-H	1,082	-	-	-	-
C=O	1,249 (1,241)	-	-	-	-
C(=O)-O	1,458	-	-	-	-

Table S5. The theoretical values of the HOMO-LUMO energies for the systems studied.

System	E (HOMO), a.u.	E (LUMO), a.u.	E (gap), eV
CO2	-0,36997	0,02992	10,88
MHA	-0,20703	0,03593	6,61
DHA	-0,20309	0,03825	6,57
THA1	-0,20320	0,03296	6,43
$MHAH^+$ - $MHACOO^-$ (2:1 for HA:CO_2)	-0,20893	-0,01387	5,31
$DHAH^+$ - $DHACOO^-$ (HA:CO_2 2:1)	-0,20749	-0,01231	5,31
$THA1H^+$ - $THA1COO^-$ (HA:CO_2 2:1)	-0,20745	-0,01739	5,17
$MHAH^+$ - $MHACOO^-$ (3:2 for HA:CO_2)	-0,20738	-0,01655	5,19
$DHAH^+$ - $DHACOO^-$ (HA:CO_2 3:2)	-0,20716	-0,01289	5,29
